# Radiation-induced rescue effect

**DOI:** 10.1093/jrr/rry109

**Published:** 2019-01-08

**Authors:** Kwan Ngok Yu

**Affiliations:** 1 Department of Physics, City University of Hong Kong, Tat Chee Avenue, Kowloon Tong, Hong Kong; 2 State Key Laboratory in Marine Pollution, City University of Hong Kong, Tat Chee Avenue, Kowloon Tong, Kowloon, Hong Kong

**Keywords:** radiation-induced rescue effect, radiation-induced bystander effect, bilateral bystander responses, reciprocal bystander effect

## Abstract

Radiation-induced rescue effect (RIRE) refers to the phenomenon in which detrimental effects in targeted irradiated cells are reduced upon receiving feedback signals from partnered non-irradiated bystander cells, or from the medium previously conditioning these partnered non-irradiated bystander cells. For convenience, in the current review we define two types of RIRE: (i) Type 1 RIRE (reduced detrimental effects in targeted cells upon receiving feedback signals from bystander cells) and (ii) Type 2 RIRE (exacerbated detrimental effects in targeted cells upon receiving feedback signals from bystander cells). The two types of RIRE, as well as the associated mechanisms and chemical messengers, have been separately reviewed. The recent report on the potential effects of RIRE on the traditional colony-formation assays has also been reviewed. Finally, future priorities and directions for research into RIRE are discussed.

## INTRODUCTION

Radiation-induced rescue effect (RIRE) refers to the phenomenon in which detrimental effects in targeted irradiated cells are reduced upon receiving feedback signals from partnered non-irradiated bystander cells, or from the medium previously conditioning these partnered non-irradiated bystander cells. RIRE was discovered in 2011 [1] and is closely related to the radiation-induced bystander effect (RIBE). RIBE refers to the phenomenon in which non-irradiated bystander cells respond as if they themselves have been irradiated upon being partnered with targeted irradiated cells or upon being immersed in the medium having previously conditioned the targeted irradiated cells. There are already many excellent reviews on RIBE in the literature [2–8], so it is stressed at the outset that the present paper does not aim to provide a review on RIBE. RIRE was previously reviewed by Lam *et al.* [9] in the context of its effects on the efficacy of conventional radioimmunotherapy (RIT). Since then, a number of important advances have been made in RIRE research, which will be highlighted in the present review.

First, when α-particle–induced RIRE was discovered in co-cultured normal human lung fibroblast (NHLF) cells and human cervical cancer (HeLa) cells in 2011 [1], RIRE was defined as the reduction in detrimental effects in the targeted cells upon receiving feedback signals from bystander cells. For example, the levels of p53-binding protein 1 (53BP1) and micronucleus (MN) formation in α-particle–irradiated cells, the surviving fraction from colony-formation assays, and the number of annexin V–positive (FL1-H) apoptotic cells upon α-particle irradiation were reduced in the presence of co-cultured bystander cells. Interestingly, however, in 2016, Fu *et al.* [10, 11] revealed another type of RIRE, in which the detrimental effects were exacerbated in the targeted cells upon receiving feedback signals from bystander cells. It is still not certain whether these two types of RIRE are merely different manifestations of the same phenomenon, but it is expected that identification of the underlying mechanisms will help clarify the situation. For convenience, in the current review we refer to these two types of RIRE as: (i) Type 1 RIRE (reduced detrimental effects in targeted cells upon receiving feedback signals from bystander cells) and (ii) Type 2 RIRE (exacerbated detrimental effects in targeted cells upon receiving feedback signals from bystander cells). In other words, Type 1 RIRE is the type unveiled in 2011 [1], whereas Type 2 RIRE is the type reported in 2016 [10, 11]. Notwithstanding, Kong *et al.* [12] remarked that the combination of irradiated/non-irradiated cell types in the experiments involved in revealing Type 2 RIRE was different from that used in the studies that showed Type 1 RIRE. Type 1 RIRE will first be reviewed in the section on ‘Type 1 rescue effect’, and Type 2 RIRE (together with the associated mechanisms and chemical messengers) will then be reviewed in the section on ‘Type 2 rescue effect’.

Second, subsequent to the discovery of RIRE in 2011 [1] (now referred to as Type 1 RIRE), various research groups further succeeded in demonstrating RIRE using different cell lines and different types of ionizing radiations. The previous review [9] remarked that the significance of RIRE varied according to the types of the irradiated and bystander cells, the biological end points and the radiation dose, and advocated that studies on the radiation dose response would be relevant for understanding and for application of RIRE. Now that Type 2 RIRE has been reported in experiments involving α particles [10, 11], but not yet in experiments using other types of ionizing radiations such as photons and protons, it would be pertinent to study in future whether Type 2 RIRE could also be induced by other types of ionizing radiations, or more generally whether the nature of RIRE would depend on the type of ionizing radiation used. Accordingly, the various studies on RIRE will be reviewed in the section on ‘Other studies confirming Type 1 rescue effect’ according to the types of ionizing radiations employed, including photons [12–14], alpha particles [15–17] and protons [18–20]. The Type 1 RIRE reported [12, 19, 20] since the previous review [9] will be reviewed here in the section on ‘Other studies confirming Type 1 rescue effect’. However, the Type 1 RIRE reported in references [13–18] were previously reviewed [9], so these will only be briefly described. The studies reported in references [10, 11] referred to Type 2 RIRE and will be reviewed in the section on ‘Type 2 rescue effect’.

Third, studies on mechanisms and chemical messengers are important for understanding RIRE. He *et al.* [17] demonstrated that Type 1 RIRE was mediated by cyclic adenosine monophosphate (cAMP) through a membrane signaling pathway, and Lam *et al.* [15, 16] revealed that the rescue signal responsible for Type 1 RIRE activated the nuclear factor κB (NF-κB) response pathway in the irradiated cells. These mechanisms have been reviewed in reference [9]. Nitric oxide (NO) was also found capable of stimulating or inhibiting NF-κB activity [21–23], and mechanisms underlying radiation-induced, NO-mediated bystander effects [24–31] could also be involved in Types 1 and 2 RIRE. More recently, Kong *et al.* [12] proposed that Type 1 RIRE was initiated by bystander factors released from irradiated cells, which induced autophagy and activated the signal transducer and activator of transcription 3 (STAT3) to produce interleukin 6 (IL-6) in bystander cells, and the secreted IL-6 activated the NF-κB pathway in irradiated cells. This mechanism will be reviewed in more detail in the section on ‘Mechanisms underlying Type 1 RIRE’ together with a brief description of previously identified mechanisms.

Fourth, Adrian *et al.* [32] recently reported interesting potential effects of RIRE on traditional colony-formation assays, which were first introduced by Puck and Marcus in 1956 [33]. Adrian *et al.* [32] found that when the number of irradiated cells increased, the corresponding surviving fraction also increased, and thus proposed the presence of a ‘cell-density–dependent rescue-like effect’ or in short a ‘cell density effect’ in the assay. These effects will be reviewed in the section on ‘Effects of RIRE on traditional colony-formation assays’.

The last section is a discussion about priorities and directions for further research into RIRE.

## TYPE 1 RESCUE EFFECT

As mentioned in the Introduction, RIRE was discovered in 2011 [1] and refers to reduced detrimental effects in targeted cells upon receiving feedback signals from bystander cells. This type of RIRE is referred to as Type 1 RIRE in the present review, to distinguish it from another type of RIRE (referred to as Type 2 RIRE in the present review)—exacerbated detrimental effects in targeted cells upon receiving feedback signals from bystander cells. We note that, back in 2007, Mackonis *et al.* [34] observed increased survival of irradiated cells when they were partnered with cells irradiated with a low dose, and they referred to this phenomenon as the ‘Type 3’ bystander effect. Notably, Chen *et al.* [1] highlighted the difference between RIRE and ‘Type 3’ bystander effect: the bystander cells were non-irradiated in RIRE, whereas they were irradiated to create the ‘type 3’ bystander effect.

## TYPE 2 RESCUE EFFECT

As mentioned in the Introduction, Fu *et al.* [10, 11] uncovered a different type of RIRE, which displayed exacerbated detrimental effects in targeted cells upon receiving feedback signals from bystander cells; this is referred to as Type 2 RIRE in the present review. The authors revealed Type 2 RIRE in targeted human bronchial epithelial cells (Beas-2B) irradiated with α particles when the targeted cells were co-cultured with non-irradiated human macrophage U937 cells (derived from histiocytic lymphoma). Fu *et al.* [10] also proved that the detriment was exacerbated in irradiated Beas-2B cells through activation of mitogen-activated protein kinases (MAPKs) and NF-κB pathways in the bystander U937 cells. Fu *et al.* [11] further examined the Type 2 RIRE in targeted Beas-2B cells irradiated with α particles, when the targeted cells were co-cultured with non-irradiated U937 cells. The authors reported that TNF-α and interleukin 8 (IL-8) were upregulated in the U937 cells, which was relayed on the activated extracellular signal–regulated kinases (ERKs) and p38 pathways in the Beas-2B cells and was also due to the activated NF-κB pathway in the U937 cells.

Nevertheless, Kong *et al.* [12] cautioned that the combination of irradiated/non-irradiated cell types in these experiments was different from that used in most studies that showed Type 1 RIRE. Kong *et al.* [12] proposed that the cell type (regardless of whether irradiated or non-irradiated) was another crucial factor in determining its role in rescuing partnered cells or in being rescued by partnered cells, and proposed that both irradiated cells and cancer cells be classified as ‘stressed’ cells that sought to be rescued. In most studies showing Type 1 RIRE, either the same cell lines were used for irradiated and bystander cells, or in the cases where cancer cells and non-transformed cells were involved, the cancer cells were irradiated whereas the non-transformed cells were non-irradiated. However, in the experiments of Fu *et al.* [10, 11], the non-transformed (Beas-2B) cells were irradiated, whereas the cancer (U937) cells were non-irradiated. As such, it is not yet certain whether Types 1 and 2 RIRE are merely different manifestations of the same phenomenon, but it has been suggested that identification of the underlying mechanisms will help clarify the situation. Kong *et al.* [12] also pointed out that such a conjecture aligns with observations from some previous studies. Widel *et al.* [13] confirmed RIRE in irradiated human melanoma (Me45) cells co-cultured with non-irradiated non-transformed normal human dermal fibroblasts (NHDFs), but revealed that non-irradiated Me45 cells did not rescue co-cultured irradiated NHDF cells. Desai *et al.* reported that for irradiated lung adenocarcinoma (A549) cells, bystander human lung normal fibroblast (WI38) cells provided a much stronger rescue effect than bystander A549 cells [18].

## OTHER STUDIES CONFIRMING TYPE 1 RESCUE EFFECT

Subsequent to the discovery of Type 1 RIRE in 2011 [1], various research groups further succeeded in demonstrating RIRE using different cell lines and different types of ionizing radiations. As explained in the Introduction, Type 2 RIRE was reported in Beas-2B cells irradiated with α particles [10, 11]. Interestingly, however, as of today, Type 2 RIRE has not yet been identified in experiments using other types of ionizing radiations such as photons and protons. It would be pertinent to study in future whether Type 2 RIRE could also be induced by other types of ionizing radiations, or more generally whether the nature of RIRE depends on the type of ionizing radiation employed. In fact, to better understand the RIRE derived from different types of ionizing radiations, it might be necessary to perform separate experiments and compare the results obtained using different types of ionizing radiations. The various studies are reviewed here according to the types of ionizing radiations employed.

### Photon (X/γ-ray)-induced RIRE

Widel *et al.* [13] observed Type 1 RIRE in targeted human melanoma (Me45) cells irradiated with 6 MV X-rays, when these targeted cells were co-cultured with non-irradiated NHDF cells, revealed through reduced micronuclei (MN) formation and apoptosis. The authors also reported that non-irradiated Me45 cells failed to rescue targeted Me45 cells or targeted NHDF cells. Pereira *et al.* [14] confirmed Type 1 RIRE in targeted embryonic zebrafish fibroblast (ZF4) cells irradiated using ^137^Cs γ-rays when the targeted cells were partnered with non-irradiated ZF4 cells, revealed through fewer γ-H2AX foci. Kong *et al.* [12] demonstrated Type 1 RIRE in targeted HeLa cells irradiated with 200 kV X-rays with a 2 mm Al filter, when they were partnered with non-irradiated HeLa cells, particularly when autophagy was pre-induced in the non-irradiated HeLa cells before partnering. Adrian *et al.* [32] proposed that RIRE could potentially have an influence on the traditional colony-formation assays, first introduced by Puck and Marcus in 1956 [33], using the human prostate cancer DU-145 cell line and the human melanoma MM576 cell line, and by using 120 kV X-rays with a 2 mm Al filter. However, as explained in the section on ‘Effects of RIRE on traditional colony-formation assays’ below, it was not yet certain whether these effects on colony-formation assays were indeed explained by the RIRE.

### Alpha-particle–induced RIRE

He *et al.* [17] confirmed Type 1 RIRE in targeted human macrophage U937 cells irradiated with α particles, when the targeted cells were co-cultured with non-irradiated hepatocyte HL-7702 cells, revealed through reduced MN formation. The authors proved that bystander cells communicated cAMP to targeted cells through a membrane signaling pathway. Lam *et al.* [15, 16] confirmed Type 1 RIRE in targeted human cervical cancer HeLa cells as well as in mouse embryo fibroblast NIH/3T3 cells irradiated with α particles, when the targeted cells were co-cultured with the same cell lines that were non-irradiated, revealed through reduced numbers of 53BP1 foci/cell and through increased phosphorylated NF-κB (p-NF-κB) expression. Type 1 RIRE was also demonstrated in both targeted cell lines upon treatment with the conditioned medium (CM), which had conditioned their bystander counterparts previously partnered with irradiated cells from the same cell lines. The authors proved the presence of a rescue signal in the CM, and reported that RIRE was activated through the NF-κB pathway in the irradiated cells. The authors also showed that induction of RIRE critically depended on the ratio between (number of non-irradiated bystander cells) and (number of irradiated cells). A novel method was also designed for preparing the CM that only contained rescue signals from bystander cells (without bystander signals from irradiated cells).

### Proton-induced RIRE

Desai *et al.* [18] and Kobayashi *et al.* [20] confirmed Type 1 RIRE in targeted lung adenocarcinoma (A549) cells irradiated with 3.4-MeV microbeam protons when the targeted cells were co-cultured with non-irradiated human lung normal fibroblast (WI38) cells, revealed through γ-H2AX foci fluorescence intensity per nucleus. Liu *et al.* [19] studied proton-induced Type 1 RIRE between co-cultured cancer stem-like cells (CSCs) and non-stem-like cancer cells (NSCCs) of the human fibrosarcoma HT1080 cell line through the level of 53BP1 accumulation in the cells. Targeted cells were irradiated with 3.4-MeV microbeam protons. The authors revealed that bystander CSCs rescued targeted NSCCs but not targeted CSCs, whereas bystander NSCCs did not rescue targeted CSCs or NSCCs.

## MECHANISMS UNDERLYING TYPE 1 RIRE

### Involvement of cAMP

Upon demonstrating RIRE between co-cultured α-particle–irradiated human macrophage U937 cells and bystander non-irradiated HL-7702 hepatocyte cells, He *et al.* [17] revealed that cAMP communicated through a membrane signaling pathway from the bystander cells to the irradiated cells, alleviating the radiation damages in the latter, and that cAMP diminution in irradiated cells upon irradiation was compensated by cAMP from bystander cells. The authors thus hypothesized that RIRE was mediated by cAMP communicated from bystander cells to irradiated cells. Lam *et al.* [15, 16] remarked that involvement of cAMP in RIRE was compatible with activation of the NF-κB pathway (see subsection on ‘Involvement of NF-κB pathway activation’ below).

### Involvement of NF-κB pathway activation

While demonstrating RIRE in α-particle–irradiated HeLa and NIH/3T3 cells (when the targeted cells were co-cultured with the same cell lines that were non-irradiated), Lam *et al.* [15, 16] revealed that RIRE was activated through the NF-κB pathway in the irradiated cells. The authors commented that involvement of NF-κB pathway activation aligned with the following observations: (i) RIRE involved soluble factors but not gap junction intercellular communication (GJIC), even in the presence of the latter [18]; (ii) RIRE involved cAMP communication from bystander cells to irradiated cells [17]; (iii) RIRE was associated with a decrease in the ROS level in the irradiated cells [13]. Although Lam *et al.* [15, 16] suggested tumor necrosis factor-α (TNF-α) as a potential soluble molecule that could activate the NF-κB pathway in the irradiated cells, this did not preclude involvement of other soluble molecules with a similar function. In fact, Kong *et al.* [12] established that IL-6 was involved in communicating RIRE (see the subsection on ‘Induction of autophagy and IL-6 secretion in bystander cells’ below).

### Involvement of NO

There is also evidence that NO could be potentially involved in the mechanisms for Type 1 RIRE. The involvement of NO in the bystander effect was extensively studied by Matsumoto and colleagues, and it could also be involved in Types 1 and 2 RIRE. In particular, the radiation-induced, NO-mediated ‘protective bystander effect’ [24–29] could be involved in Type 1 RIRE, whereas the ‘radiation-induced, NO-mediated bystander cell killing’ [30] and the ‘radiation-induced, NO-mediated bystander reduction of spontaneous mutations’ [31] could be involved in Type 2 RIRE. Interestingly, previous studies showed that NO could both stimulate and inhibit NF-κB activity, and various mechanisms were proposed that might explain the different manifestations (i.e. Types 1 and 2) of RIRE. On one hand, the NF-κB activity could be stimulated through S-nitrosation of p21^ras^ and thus Ras in response to oxidative stress [21], where Ras was implicated in NF-κB stimulation [22, 23]. NO has also been demonstrated to sustain nuclear translocation of RelA (p65) and thus a persistent activation of NF-κB [35]. On the other hand, the NF-κB activity could be inhibited through both S-nitrosation of the p50 subunit of NF-κB [36] and enhancement of IκB production [37]. As such, modulation of the NF-κB activity depends on the cell type and experimental conditions, and might depend on local concentrations of NO [38] or its redox end-products [39]. As discussed in the preceding subsection, stimulation of NF-κB activity has been proposed to be involved in the RIRE in irradiated cells, so the manifestations (i.e. Types 1 and 2) might also depend on the cell type, as well as the local concentrations of NO [38] or its redox end-products [39]. Further studies are needed to investigate such possibilities.

### Induction of autophagy and IL-6 secretion in bystander cells

Kong *et al.* [12] explored the similarity between RIRE and metabolic crosstalk between cancer cells and non-transformed cells in tumor microenvironments to effect the so-called ‘metabolic cooperation’ (e.g. see review in Ref. [40]), in which non-transformed cells are prompted to supply nutrients to support the survival and growth of cancer cells [41–47]. As part of the nutrient acquisition strategies, the oncogenes and tumor suppressors in cancer cells could have been mutated to grant these cells autonomy over nutrient uptake, as well as to increase the availability of precursors for macromolecular synthesis [40]. A unified scheme was proposed by Kong *et al.* [12] to involve generalized ‘stressed’ cells (including both irradiated cells as well as nutrient-depleted cancer cells) and generalized ‘bystander’ cells (including those non-irradiated cells partnering with the irradiated cells as well as non-transformed cells metabolically cooperating with nutrient-depleted cancer cells).

Kong *et al.* [12] studied RIRE in X-ray–irradiated HeLa cells and investigated the induction of autophagy and IL-6 secretion in the bystander cells. The authors first revealed autophagy induction in irradiated cells (speculated to supply molecules required for cell-repair enhancement) in the absence of bystander non-irradiated cells. When bystander non-irradiated cells were partnered with the irradiated cells, autophagy was induced in the non-irradiated cells, while autophagy accumulation in irradiated cells was significantly alleviated, which hinted that the autophagy induced in non-irradiated cells supported the irradiated cells. Reduction in autophagy accumulation in irradiated cells was more significant if autophagy was pre-induced in the non-irradiated cells before partnering. The RIRE in the irradiated cells was also enhanced, while the RIBE displayed in the bystander cells decreased with pre-induction of autophagy in the bystander cells.

The authors went on to reveal the IL-6 secretion by these bystander non-irradiated cells, especially those with pre-induced autophagy, when they were cultured in the medium having previously conditioned irradiated HeLa cells. These results suggested that IL-6 could help mitigate damages induced by ionizing radiations. At the same time, the authors also observed that autophagy pre-induction in non-irradiated cells would enhance IL-6 secretion in these non-irradiated cells as well as RIRE in irradiated cells. Judging from these observations, the authors proposed that RIRE could be interpreted as a metabolic cooperation process. Specifically, this process was initiated by bystander factors released from irradiated cells, which induced autophagy and IL-6 secretion (speculated to be produced through activation of STAT3 in bystander non-irradiated cells), and was finally manifested in activation of the NF-κB pathway in irradiated cells by IL-6 secreted by the non-irradiated cells.

Kong *et al.* [12] also proposed that the existence of Types 1 and 2 RIRE might be explained through the metabolic cooperation between generalized ‘stressed’ cells and generalized ‘bystander’ cells. In the revelation of Type 2 RIRE, Fu *et al.* [10, 11] irradiated Beas-2B cells derived from normal bronchial epithelium obtained from autopsy of non-cancerous individuals, and employed human macrophage (U937) cells derived from histiocytic lymphoma as bystander non-irradiated cells. Considering the stress due to the cell types alone, it is possible that the ‘stressed’ U937 cells secreted bystander factors to induce autophagy in the non-transformed ‘bystander’ Beas-2B cells to provide help in a similar manner to that of the induction of autophagy by ‘stressed’ pancreatic cancer cells in the neighboring non-cancerous pancreatic stellate cells (to release alanine to help the pancreatic cancer cells survive). On the contrary, however, considering the stress due to the irradiation alone, the irradiated ‘stressed’ Beas-2B cells could secrete bystander factors to induce autophagy in the non-irradiated ‘bystander’ U937 cells to supply molecules required for cell-repair enhancement in the Beas-2B cells. In the end, the cells controlled by their partners to undergo enhanced autophagy instead of controlling their partners to provide support would exhibit exacerbated effects compared with the expected effects from its initial sustained stress, which could provide an explanation for the behavior of the Beas-2B cells in this case. This conjecture could also explain the findings from a number of previous studies. While Widel *et al.* [13] confirmed RIRE in irradiated human melanoma (Me45) cells co-cultured with non-irradiated NHDFs, the authors revealed that non-irradiated Me45 cells did not rescue co-cultured irradiated Me45 cells or co-cultured irradiated NHDF cells. These results could be understood if the Me45 cells always dictated the metabolic crosstalk in their capacity as ‘stressed’ cells (due to the cell type). Desai *et al.* also revealed that for irradiated lung adenocarcinoma (A549) cells, bystander human lung normal fibroblast (WI38) cells induced a much stronger rescue effect than bystander A549 cells [18], which again suggested the dictation of the A549 cells in the metabolic crosstalk. Nevertheless, despite these studies showing unexpected RIRE in X-ray– and proton-irradiated non-transformed cells when they were partnered with non-irradiated cancer cells, the exacerbated Type 2 RIRE did not show up as that displayed in Beas-2B cells irradiated with α particles [10, 11]. Whether the difference was due to the use of different types of ionizing radiations remains to be explored in future studies using different types of ionizing radiations.

### Scheme for activation of NF-κB pathway in RIRE

**Fig. 1.**
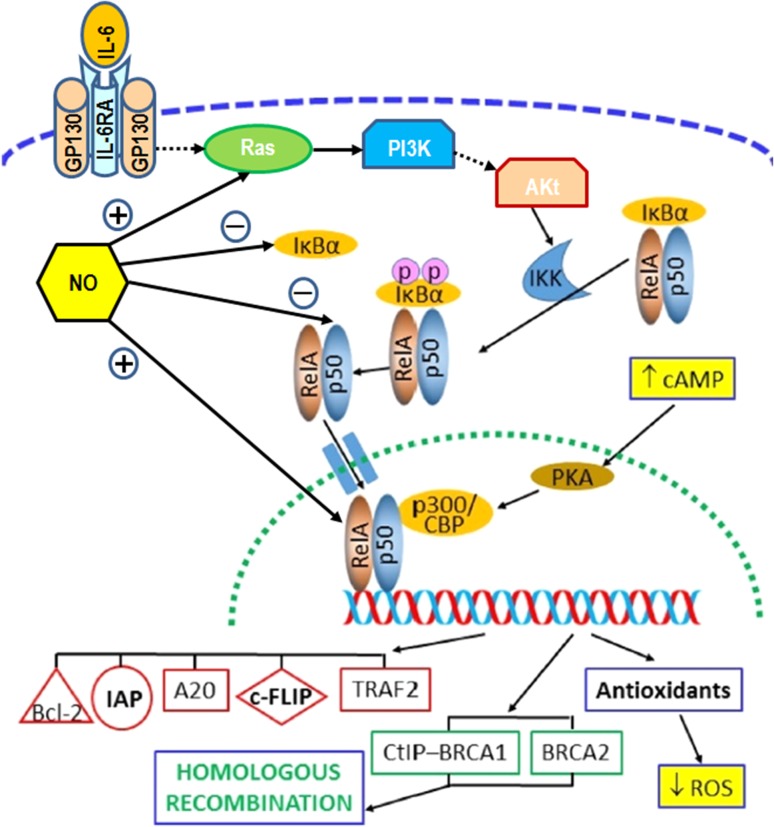
Schematic diagram to illustrate the activation of the nuclear factor-κB (NF-κB) pathway in the radiation-induced rescue effect (RIRE). The diagram also incorporates observations from previous research (in yellow boxes), namely, the increase in the level of cyclic adenosine monophosphate (cAMP) and the decrease in the level of ROS in irradiated cells, as well as the involvement of NO in radiation-induced bystander effects. The prototypical heterodimer with RelA (p65) and p50, which are NF-κB family members comprising, has been used for illustration. Blue dashed line: cell membrane; green dotted line: nuclear envelope; dotted arrows: multiple steps involved; IL-6: interleukin 6; IL-6RA: interleukin 6 receptor alpha; GP130: glycoprotein 130; PI3K: phosphatidylinositol 3-kinase; Akt: Protein kinase B (PKB); IKK: IκB-kinase; PKA: protein kinase A; CBP: CREB-binding protein; CREB: cAMP response element–binding protein; bcl-2: B-cell lymphoma 2 protein; IAPs: inhibitors of apoptosis protein; A20: A20 zinc finger protein; c-FLIP: cellular FLICE-like inhibitory protein; FLICE: FADD-like interleukin-1β–converting enzyme; FADD: Fas-associated protein with death domain; TRAF2: TNF receptor–associated factor 2; CtIP: C-terminal binding protein (CtBP)-interacting protein; BRCA1: breast cancer type 1 susceptibility protein; BRCA2: breast cancer type 2 susceptibility protein.

Figure 1 is a schematic diagram illustrating the activation of the NF-κB pathway in RIRE. Figure 1 also incorporates observations from previous research on RIRE, namely, increase in the level of cAMP [17] and decrease in the level of reactive oxygen species (ROS) [13] in irradiated cells, as well as involvement of NO in RIBE [21–31]. The prototypical heterodimer with RelA (p65) and p50, which were NF-κB family members comprising, were used for illustration.

## EFFECTS OF RIRE ON TRADITIONAL COLONY-FORMATION ASSAYS

In 2018, Adrian *et al.* reported very interesting potential effects of RIRE on the traditional colony-formation assays first introduced by Puck and Marcus in 1956 [32]. Through uniformly irradiating the human prostate cancer DU-145 cell line and the human melanoma MM576 cell line by 120 kV X-rays with a 2 mm Al filter, the authors found that when the number of irradiated cells increased, the corresponding surviving fraction also increased, and thus proposed the presence of a ‘cell-density–dependent rescue-like effect’ or in short a ‘cell density effect’ in the assay. This finding presented a challenge to the colony-formation assay, because in practice a larger number of irradiated cells would be employed for studying the effects from larger radiation doses to maintain a manageable number of colonies. Adrian *et al.* [32] also found that when cells were irradiated with modulated beams so that the irradiated cells could communicate with more non-irradiated cells during the colony-formation period, the in-field surviving fraction also increased, which was also explained in terms of RIRE. The authors also suggested that the cell density effect could partly explain the experimental results for RIBE obtained using modulated beam irradiation and colony-formation assays [48–54]. However, it appears that RIRE only refers to the effect on irradiated cells of non-irradiated bystander cells ever since RIRE was identified [1]. As described in the section on ‘Type 1 rescue effect’ above, Chen *et al.* [1] clearly cautioned about the difference between Type 1 RIRE in which the bystander cells were non-irradiated, and the ‘Type 3’ bystander effect reported by Mackonis *et al.* [34], in which the bystander cells were irradiated. In fact, to ensure compliance with the definition of Types 1 and 2 RIRE, special attention was paid to make sure that the irradiated cell population should not be ‘contaminated’ with non-irradiated cells [55] to avoid potentially ambiguous ‘resultant’ radiobiological effects. Apparently, the involved mechanisms and the chemical messengers should be identified before a conclusion can be made on the similarity between Types 1 and 2 RIRE, and Type 3 bystander effect.

## DISCUSSION

The current paper reviewed the RIRE between targeted irradiated cells and non-irradiated bystander cells. Type 1 RIRE was previously reviewed by Lam *et al.* [9] in the context of its potential effects on the efficacy of RIT. Since then, a number of important advances have been made in RIRE research, which were reviewed above. However, RIRE is still far from being adequately understood, and many more investigations are needed to help elucidate the phenomenon and to help put it into real-life applications.

In particular, observation of Type 2 RIRE, which was apparently different from the RIRE first reported in 2011 [1] (referred to as Type 1 RIRE in the present review), and as of today only known through α-particle irradiation experiments, added to the complexity of the phenomenon [10, 11]. Nevertheless, Kong *et al.* [12] remarked that the combination of irradiated/non-irradiated cell types in these experiments was different from those used in other studies that showed Type 1 RIRE. On the other hand, the effects of RIRE on traditional colony-formation assays reported by Adrian *et al.* [32] were also intriguing. Adrian *et al.* [32] suggested that the effects could partly explain the experimental results for RIBE obtained using modulated beam irradiation and colony-formation assays [48–54]. However, when RIRE was first reported [1], the authors explicitly pointed out that the bystander cells were non-irradiated in RIRE, but these cells were irradiated in the ‘Type 3’ bystander effect reported by Mackonis *et al.* [34]. Apparently, the mechanisms involved and the chemical messengers for RIRE should be carefully examined before conclusions can be made on the difference between Types 1 and 2 RIRE, on the similarity between Type 1 RIRE and the ‘Type 3’ bystander effect, and on the potentially different RIRE induced by different types of ionizing radiation.

Radiotherapy has been playing a significant role in cancer treatment in the past decades. At the time of its discovery, it was already recognized that RIRE would have far-reaching consequences on the treatment outcome of radiotherapy, particularly noting that non-irradiated normal cells could rescue irradiated cancer cells [1]. There are two key issues in enabling better understanding or predicting of the treatment outcome of chosen radiotherapy procedures, i.e. establishment of the dose responses for RIRE, and exploration of the mechanisms underlying RIRE. The potential effects of radiation dose on RIRE might be inferred from previous research results. For example, Widel *et al.* [13] did not detect significant (despite finding an indication of) RIRE in irradiated fibroblasts by non-irradiated fibroblasts. This was in contrast to the results of Chen *et al.* [1], who observed the RIRE between NHLF cells, and the results of Pereira *et al.* [14] who observed the RIRE between irradiated and non-irradiated embryonic zebrafish fibroblast (ZF4) cells. Although the discrepancies might be attributed to the different ionizing radiations and/or different cell lines, they could also be explained by the different doses employed. While Widel *et al.* [13] used 2 or 4 Gy of 6 MV X-rays for irradiation, Chen *et al.* [1] used 20 cGy or 40 cGy of alpha-particle doses, and Pereira *et al.* [14] used 70 mGy or 550 mGy of gamma-ray doses from a ^137^Cs gamma irradiator.

On the other hand, successfully unveiling the mechanisms underlying RIRE will enable development of drugs and/or alternative treatment procedures to mitigate or exploit RIRE to improve the treatment outcome in patients. This task in fact echoes the general goal of identifying the mechanisms underlying RIBE. For example, Mothersill and Seymour [56] remarked that ‘bystander effects may be harnessed to produce a new generation of anti-cancer drugs’. In 2017, Peng *et al.* reported their finding that cysteine protease cathepsin B mediated RIBE in *Nature* [57]. The corresponding report in The ASCO (American Society of Clinical Oncology) Post [58] reiterated that ‘Ultimately, researchers hope (that the precise mechanism behind RIBE) could lead to medication patients could take before radiation treatment to mitigate radiation-induced bystander effect.’ The same ASCO Post [58] also quoted the lead author of the *Nature* paper: ‘Dr. Xue hoped to work with other researchers in the future to identify other RIBE factors and mechanisms and help develop drugs that inhibit them.’

Kong *et al.* [12] also discussed the potential benefits if RIRE and metabolic cooperation were found to have a common origin. For cancers involving metabolic crosstalk between cancer cells and non-transformed cells in tumor microenvironments, such as pancreatic cancers, Kong *et al.* [12] proposed that inhibition of secretion of relevant bystander factors, although many of which not yet known, could potentially provide alternative therapy methods. On the other hand, for cancers involving RIRE, Kong *et al.* [12] proposed that inactivation of autophagy pathways in non-irradiated cells might potentially lower the resistance of cancer cells, thereby enhancing the efficacy of the employed radiotherapy module.

The above discussion highlighted the important roles of mechanisms and chemical messengers in understanding RIRE, and in mitigating or exploiting RIRE in real life applications. Unfortunately, studies on mechanisms and chemical messengers are still scarce. Lam *et al.* [15, 16] proposed activation of the NF-κB response pathway in the irradiated cells as the underlying mechanism for Type 1 RIRE, which could explain previous findings for Type 1 RIRE, including (i) promotion of cellular survival as well as correct repair of DNA damages, (ii) cAMP dependence [17], and (iii) modulation of intracellular ROS level in the irradiated cells [13]. On the other hand, Fu *et al.* [10] confirmed that α-particle–induced Type 2 RIRE involved activation of MAPK and NF-κB pathways in the bystander cells, and upregulation of TNF-α and IL-8 in the bystander cells, which was relayed on the activated ERK and p38 pathways in the irradiated cells and was also due to activation of the NF-κB pathway in the bystander cells. NO could also stimulate or inhibit NF-κB activity [21–23], and radiation-induced, NO-mediated bystander effects [24–31] could also be involved in Types 1 and 2 RIRE. More recently, Kong *et al.* [12] proposed that Type 1 RIRE was initiated by bystander factors released from irradiated cells, which induced autophagy and activated STAT3 to produce IL-6 in bystander cells, and the secreted IL-6 activated the NF-κB pathway in irradiated cells. Revisiting these mechanisms and chemical messengers in the irradiated and non-irradiated cells involved in Types 1 and 2 RIRE as well as Type 3 bystander effect, and using different types of ionizing radiation would be crucial to helping differentiate or unify these apparently different/similar phenomena, as well as in future development of drugs and/or alternative treatment procedures to improve the treatment outcome in cancer patients.

As reviewed above, various research groups have studied and demonstrated RIRE. However, a wide variety of irradiated and non-irradiated cells, biological end points and doses (in addition to using different ionizing radiations) were involved, which has made the results difficult to compare. A preference for future research on RIRE might be to first focus on the cell lines, biological end points and doses already studied in previous studies to facilitate comparison, unless for particular objectives that could not be achieved otherwise. The different doses will be taken care of by studying the radiation dose response for RIRE.
